# P-1758. Informatics-Genomic Pipeline for Large-Scale Extraction and Classification of AMR Genes Using CARD and AMRFinderPlus

**DOI:** 10.1093/ofid/ofaf695.1929

**Published:** 2026-01-11

**Authors:** Damani Andre, Ali Aslam, Rayven Todd, Muhammed Idris, Rafael Ponce-Terashima, Kenneth Onyedibe, Ubong Aniefiok Udoh, Philip Nwajiobi-Princewill, Ayomide Henrietta Adeyemi

**Affiliations:** Morehouse School of Medicine, Stockbridge, GA; Morehouse School of Medicine, Stockbridge, GA; Morehouse School of Medicine, Stockbridge, GA; Morehouse School of Medicine, Stockbridge, GA; Mercer University School of Medicine, Macon, Georgia; Morehouse School of Medicine, Stockbridge, GA; University of Calabar Teaching Hospital, Calabar, Cross River, Nigeria; National Hospital Abuja, Abuja, Federal Capital Territory, Nigeria; VN Karazin Kharkiv National University, Austell, Georgia

## Abstract

**Background:**

The global rise in antimicrobial resistance (AMR) poses a major threat to public health, requiring robust bioinformatic tools essential for extracting and tracking resistance genes from databases to understand AMR gene transmission and spread. Hence, this study benchmarked AMR gene extraction methods from databases such as Comprehensive Antibiotic Resistance Database (CARD) and AMRFinderPlus.

**Table 1:**
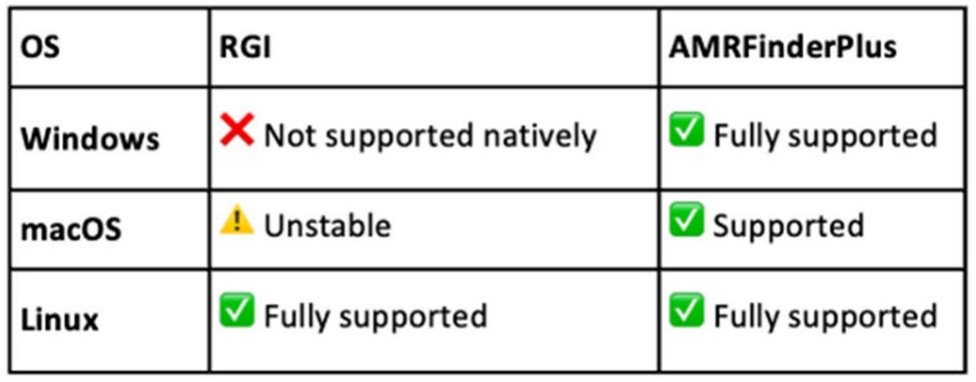
Comparison of operating systems seamlessly supported by RGI and AMRFinderPlus

**Figure 1:**
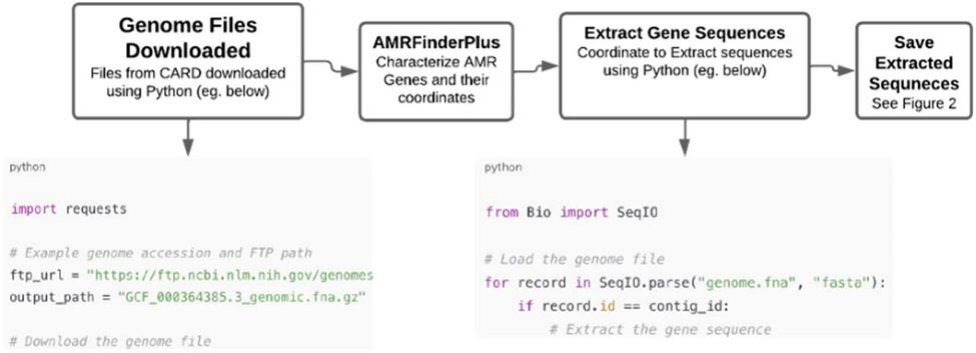
Workflow for AMR Gene Extraction from Bacterial Genomes

**Methods:**

Genomic sequences of *Klebsiella pneumoniae* (KP) and *Acinetobacter baumannii* (AB) were first downloaded using Python codes. To find and categorize the AMR genes we first used Resistance Gene Identifier (RGI), the recommended tool by CARD. We also evaluated KP and AB genome characterization using the AMRFinderPlus, a tool supported by the National Center for Biotechnology Information. We compared the ease of AMR genome characterization across Windows, macOS, and Linux systems. A custom Python pipeline was developed to automate extraction and characterization of AMR gene sequences from genome FASTA files. The workflow was designed to be capable of handling strand orientation, contig structure, and formatting differences across genomes (Figure 1).

**Figure 2:**
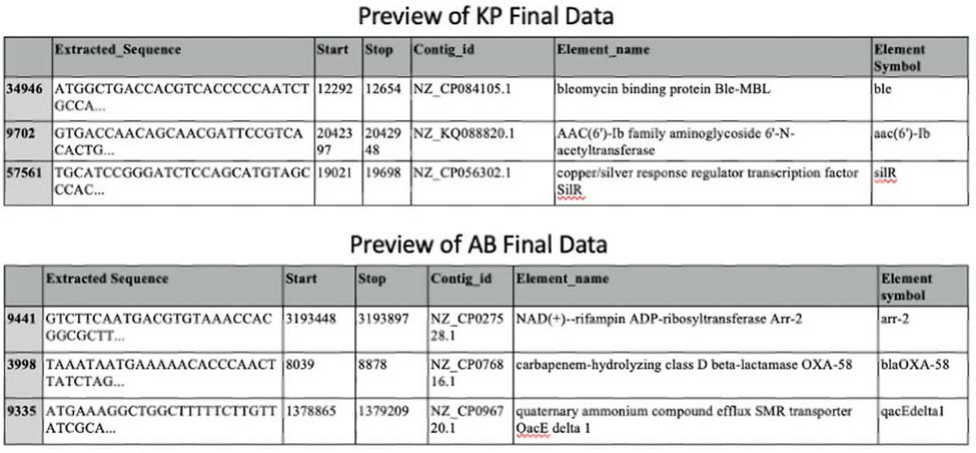
A detailed breakdown of gene coordinates, contig IDs, and gene annotations is included for representative samples

**Results:**

Resistance Gene Identifier had installation issues on macOS and windows due to strict Python version dependencies. However, it worked reliably on Windows when containerized using Docker. Also, RGI resulted in incomplete annotations, coordinate discrepancies, and file access errors. In contrast, AMRFinderPlus exhibited structured output and consistent performance across Windows, macOS, and Linux systems (Table 1). The final workflow enabled accurate extraction of resistance genes from 1123 AB isolates (10,651 Contig IDs) and 3286 KP isolates (31,406 Contig IDs). Sequences were stratified by gene name, resistance class, contig ID, start-stop location and compiled into structured datasets by Python coding. Representative annotations are shown in Figure 2.

**Conclusion:**

These findings suggest that AMRFinderPlus is a reliable solution for resistance gene characterization which could support broader applications in genomic surveillance and comparative analysis.

**Disclosures:**

All Authors: No reported disclosures

